# Investigation of Ldb19/Art1 localization and function at the late Golgi

**DOI:** 10.1371/journal.pone.0206944

**Published:** 2018-11-07

**Authors:** Jorge Y. Martínez-Márquez, Mara C. Duncan

**Affiliations:** Department of Cell and Developmental Biology, University of Michigan, Ann Arbor, Michigan, United States of America; National Heart Lung and Blood Institute, UNITED STATES

## Abstract

The arrestin-related family of proteins (ARTs) are potent regulators of membrane traffic at multiple cellular locations in the yeast *Saccharomyces cerevisiae*. Several ARTs act at multiple locations, suggesting that ARTs with well-established functions at one location may have additional, as of yet, uncharacterized roles at other locations in the cell. To more fully understand the spectrum of cellular functions regulated by ART proteins, we explored the localization and function of Ldb19/Art1, which has previously been shown to function at the plasma membrane, yet is reported to localize to the *trans-*Golgi network (TGN). We report that the C-terminal fusion of Ldb19 with GFP is functional and, as previously reported, localizes to the TGN. We further establish that Ldb19 associates with late stages of TGN maturation that are enriched in the clathrin adaptor protein complex-1 (AP-1). Additionally, we present genetic interaction assays that suggest Ldb19 acts at the late TGN in a mechanism related to that of AP-1. However, Ldb19 and AP-1 have dissimilar phenotypes in a subset of assays of membrane traffic, suggesting Ldb19 functions at the TGN are distinct from those of AP-1. Together these results indicate Ldb19 functions at the TGN, in addition to its well-established role in endocytosis.

## Introduction

Membrane traffic is a critical aspect of eukaryotic cell biology. It maintains cellular homeostasis by sending newly generated proteins to their correct locations, and by sending damaged proteins to the lysosome where they are degraded. Membrane traffic is equally important in a changing environment because it allows rapid redistribution of proteins that regulate cellular functions including small molecule transport, secretion, and signaling.

Membrane traffic is carried out in large part by cytosolic proteins that form coats [1]. Coat proteins coordinate with one another to sort the transmembrane protein cargo that will be transported away from resident proteins of the organelle, form a transport carrier, and then direct that carrier towards the correct target organelle. Different coats, which are composed of different sets or subsets of proteins, perform distinct functions by forming at different organelles, recognizing distinct cargo, and/or directing the carrier to different compartments.

Although some proteins function in a single trafficking pathway, other proteins are used in multiple pathways. A prime example of this second group of multi-use proteins is clathrin, a large hexameric protein complex that can self-assemble into a cage-like structure *in vitro* [2]. *In vivo*, clathrin is the major structural component of clathrin coats, which form at the plasma membrane and at the trans-Golgi network (TGN) and endosomes [3]. At each of these different locations, specific clathrin adaptors play pivotal roles in recognizing cargo and initiating clathrin coat assembly. In most organisms, clathrin adaptors that act at the plasma membrane and those that act at internal compartments are encoded by distinct but functionally orthologous genes [4]. However, like clathrin, some of its associated proteins act in both endocytosis and at internal compartments. Such dual functioning proteins include amphiphysin, which is important for membrane fission, and, proteins of the arrestin family, which are important for cargo sorting [5–7].

The budding yeast, *Saccharomyces cerevisiae*, contains at least 12 arrestin-related trafficking proteins (ARTs) [8,9]. ARTs are defined by the presence of an arrestin-like domain and one or more PY motifs that mediate direct interaction with the ubiquitin ligase Rsp5 [8]. Different ARTs control the traffic of different cargo in response to specific environmental cues. For example, Ldb19/Art1 mediates the endocytosis of the methionine permease Mup1 in response to methionine, while Ecm21/Art2 mediates the endocytosis of the metal ion transporter Smf1 in response to extracellular heavy metals [9,10]. ARTs can act as adaptors for Rsp5, which then ubiquitinates cargo. This ubiquitination often allows the cargo to interact directly with clathrin adaptors [11]. However, ARTs may also function independent of Rsp5 in some cases. The ART proteins Aly1/Art6 and Aly2/Art3 interact with the clathrin adaptor AP-1, raising the possibility that they may also act as physical links between cargo and the clathrin trafficking machinery, similar to the β-arrestins [12]. Moreover, mutant forms of the ART protein Rog1, which cannot bind Rsp5, attenuates G-protein-coupled receptor signaling, suggesting the ART proteins may have function in addition to their roles as Rsp5 adaptors [13].

Although all ARTs appear to act primarily in membrane traffic, our understanding of the specific function of individual ART proteins is still incomplete. In diverse yeasts, many ARTs act in endocytosis [9,14–17]. However, ARTs can have roles at internal compartments. For example, Aly1 and Aly2 localize at the trans-Golgi network (TGN) or endosomes where they control the traffic of Gap1, a general amino acid transporter [12]. Furthermore, some ART proteins act at more than one cellular location. These multifunctional ARTs include Aly1 and Aly2, which, in addition to controlling Gap1 at the TGN, also control the endocytosis of the aspartic acid/glutamic acid transporter Dip5 [18]. Similarly, the ARTs Bul1 and Bul2 act both at the TGN in biosynthetic sorting of the general amino acid permease, Gap1, and at the plasma membrane for substrate induced endocytosis of Gap1 [19]. More recently, the ART Rod1/Art4 was demonstrated to regulate sorting of the lactate permease Jen1, both at the plasma membrane and at the TGN [20]. These findings raise the possibility that even ARTs with well-established roles in endocytosis may perform additional, as of yet, uncharacterized roles at other cellular locations.

One prime candidate for an ART that may act in both endocytosis and traffic at internal compartments is Ldb19/Art1 (hereafter referred to as Ldb19). Ldb19 has clearly defined functions in the endocytosis of Mup1 in response to methionine [21,22]. However, recent studies suggest that loss of Ldb19 impairs the traffic of the arginine transporter Can1 at the TGN, not the plasma membrane, suggesting that Ldb19 may have functions at internal compartments in addition to its roles at the plasma membrane [23]. In support of the possibility that Ldb19 acts at internal compartments, a substantial fraction of fluorescently labeled Ldb19 and its *Schizosaccharomyces pombe* homolog *co-*localize with markers of the TGN [8,22,24,25]. However, it is unclear whether native Ldb19 localizes to the TGN, and, if so, whether this pool is functionally important. Here, we explored the localization of Ldb19 using a verified functional tag of Ldb19. We found that Ldb19 co-localized with markers of the TGN. Furthermore, Ldb19 co-localized strongly with and came into close proximity to the non-endocytic clathrin adaptor AP-1, further supporting the conclusion that it localizes at the TGN. We showed that Ldb19 was not required for the localization of AP-1 or other clathrin adaptors at the TGN, and that Ldb19 localization was not affected by AP-1 or other clathrin adaptors. However, Ldb19 localization depended on the function of the small GTPase Arf1. Finally, genetic interactions and assays of membrane traffic suggested Ldb19 performs functions at the TGN related to but distinct from those of AP-1. These results establish a role for Ldb19 at the TGN, in addition to its well-established function in endocytosis.

## Results

**Fig 1:**
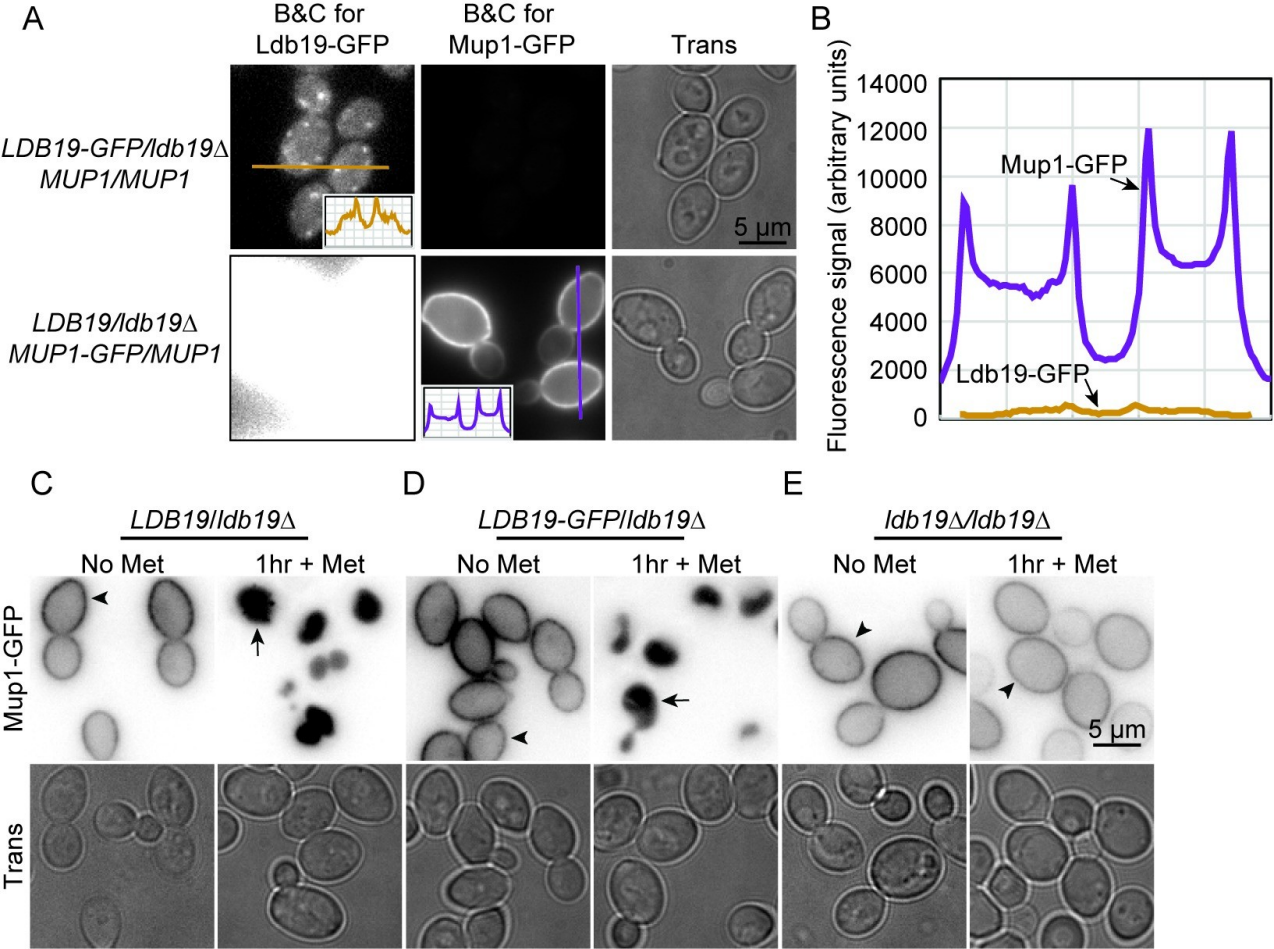
Ldb19-GFP is a functional protein. (A) Comparison of fluorescence intensity of Ldb19-GFP and Mup1-GFP. Micrographs show images collected under identical conditions but processed for optimal visualization of Ldb19 (left) or Mup1 (center). Lines indicate regions used for line-scan measurements shown in inset. (B) Line-scan measurements from A plotted together to show difference in fluorescence intensity. (C) WT/*ldb19Δ* heterozygous diploid cells expressing Mup1-GFP were imaged in the absence or 1hr after the addition of methionine, which induces endocytosis of Mup1-GFP. (D), (E) Ldb19-GFP/*ldb19Δ* heterozygous diploid or *ldb19Δ* homozygous diploid cells imaged as in A. Arrowhead indicates plasma membrane localization, arrow indicates vacuolar localization. Top row, fluorescence microscopy images with black and white values inverted; bottom row, transmitted images.

Although Ldb19 has strong effects on endocytosis, previous studies reported a significant fraction of fluorescently tagged Ldb19 was localized at internal compartments. In one high-throughput study, Ldb19 tagged at its endogenous locus co-localized with the clathrin heavy chain subunit Chc1, a marker commonly used to label the TGN [25]. Similarly, in other studies using Ldb19-GFP expressed from a plasmid, Ldb19 co-localized with the Arf-GEF Sec7, another marker commonly used to label the TGN [8,22]. These localization results are surprising given the strong endocytic phenotype caused by lack of Ldb19. However, to our knowledge, the functionality of the fluorescently tagged Ldb19 was not explored, raising the possibility that the fluorescently labeled Ldb19 was non-functional and potentially mislocalized. To test the functionality of tagged Ldb19, we monitored its function in endocytosis. To do this, we monitored methionine-induced endocytosis of Mup1-GFP in diploid cells that expressed a single copy of wild-type Ldb19 or Ldb19-GFP from its endogenous locus. In these cells, only Mup1-GFP fluorescence is clearly visible because Mup1 is expressed at 10–20 fold higher levels than Ldb19 (Fig 1A) [26,27].

In all strains tested, Mup1 was found exclusively at the plasma membrane in the absence of methionine (Fig 1C–1E). After 1hr growth in the presence of methionine, in cells expressing wild-type Ldb19 Mup1 was found exclusively in the vacuole (Fig 1C). In contrast, in cells lacking Ldb19, Mup1 was only observed at the plasma membrane (Fig 1D). In cells expressing Ldb19-GFP, Mup1-GFP was found exclusively in the vacuole at levels nearly identical to wild-type cells (Fig 1E). These data indicate that the Ldb19-GFP fusion protein is fully functional, as measured by Mup1 endocytosis.

We next explored the localization of Ldb19 in more detail. Previous studies suggested that Ldb19 localizes to internal compartments labeled by Sec7, which is commonly considered the TGN [28,29]. However, the emerging view is that the TGN is not a static organelle in yeast. It is produced from the maturation of a Golgi compartment [30]. During this maturation process, Sec7 is recruited to transitional Golgi compartments and is retained as this transitional Golgi matures into the TGN, which itself matures and eventually dissipates due to traffic to the plasma membrane, vacuole, and endosomes [31]. To monitor the localization of Ldb19 with respect to Golgi maturation, we monitored the co-localization of Ldb19 with the clathrin adaptors Gga2, Ent5, and AP-1 that define late stages of TGN maturation [32,33]. These proteins appear in a stereotypic order. Gga2 is the first adaptor apparent at the TGN and its appearance is coincident with that of Sec7. Ent5 is recruited second, and AP-1 is recruited last.

**Fig 2:**
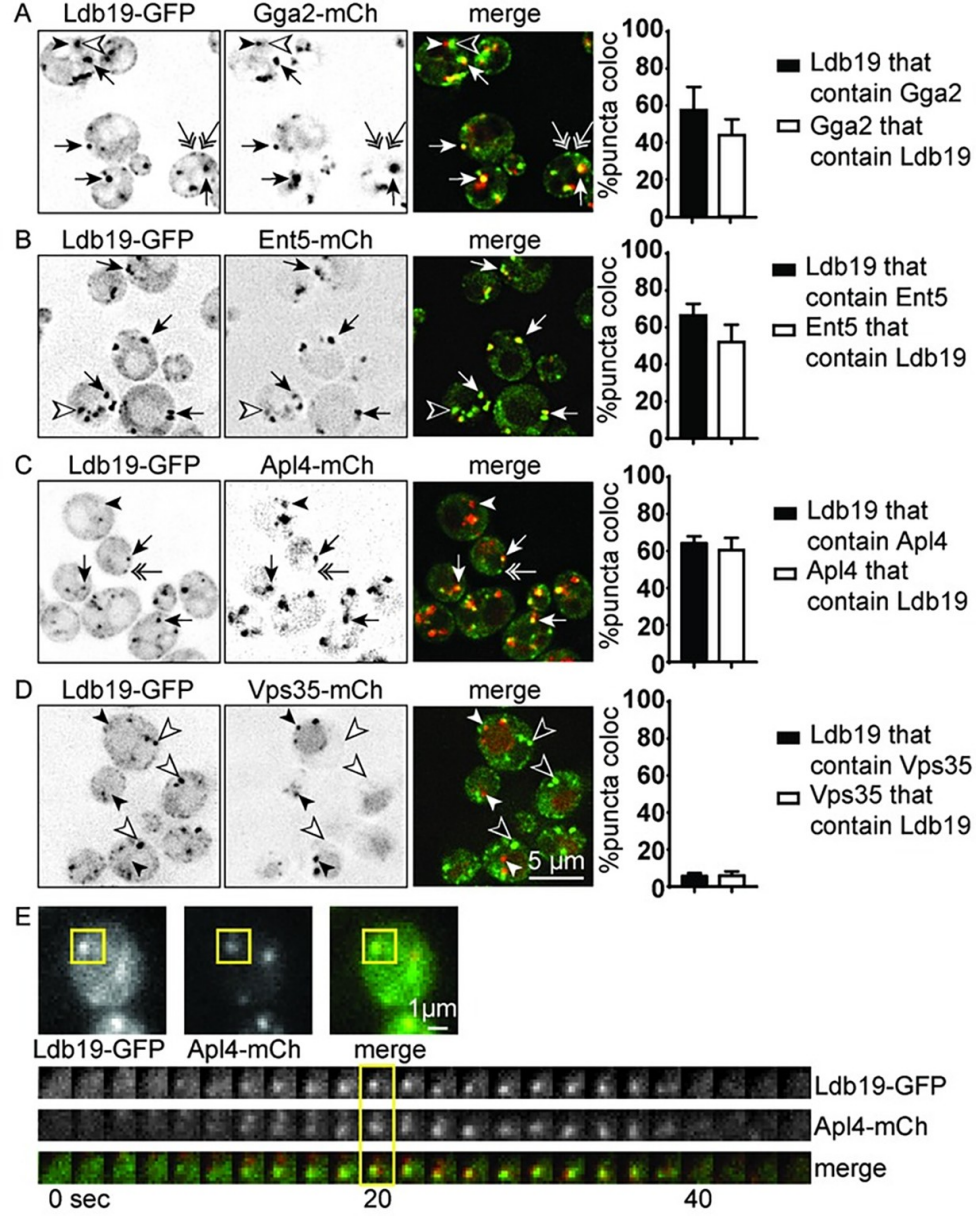
Ldb19 co-localizes with TGN clathrin adaptors. (A-D) Cells expressing indicated fusion proteins from their endogenous loci were imaged and analyzed for co-localizationArrows indicate co-localization, filled and outlined arrowheads indicate structures with only mCh or GFP, respectively, and double arrows indicate cell surface-localized Ldb19-GFP structures. Right, quantification of the percentage of Ldb19-GFP fluorescent structures that contained mCh fluorescence (black bar) and the percentage of mChfluorescent structures that contained GFP fluorescence (white bar). Error bars indicate standard deviation from the mean of three separate experiments (n = 3), each with at least 200 puncta and 70 cells. (E) Kymograph showing dynamic recruitment of Ldb19-GFP and Apl4-mCh. Top: Images of structures analyzed by live-cell microscopy. Boxed region indicate structures selected for Kymograph. Bottom: kymographs of the boxed area in the top images is shown; the boxed area indicates the time point shown above.

Using these markers for TGN maturation, we asked whether Ldb19 was preferentially associated with these late stages of TGN maturation. Consistent with its co-localization with Sec7, Ldb19 internal puncta frequently co-localized with Gga2, Ent5, and the γ-subunit of AP-1 (Apl4), but not with Vps35, a marker of endosomes (Fig 2). We also observed dim peripheral localization that was not associated with Gga2, Ent5, or AP-1, consistent with previous reports of a small pool of Ldb19 at the plasma membrane [8,22]. To quantify the co-localization between Ldb19 and mCh-tagged adaptors, we measured how many of the Ldb19-GFP fluorescent structures or foci also contained mCherry (mCh) fluorescent adaptor proteins and vice versa. We found that Ldb19 co-localized substantially with each of the adaptors. In each case nearly 60% of Ldb19 structures contained Gga2, Ent5, or AP-1., There were subtle but not statistically significant differences in the co-localization of Ldb19 with each adaptor. Ldb19 showed the least co-localization with Gga2 (58%+/- 13% SD, Fig 2A). Co-localization with Ent5 and AP-1 was slightly higher (66%+/- 6% SD and 64% +/- 3% SD, respectively; Fig 2B and Fig 2C). Moreover, the percentage of AP-1 structures that contain Ldb19 was higher than the percent of Gga2 structures that contain Ldb19 (61% +/- 6% SD versus 44% +/- 9% SD, white bars p = 0.043). These results indicate that Ldb19 is enriched during the late stages of TGN maturation, similar to AP-1 and the other clathrin adaptors.

Clathrin adaptors are dynamically recruited to the TGN. This dynamic recruitment can be monitored by time-lapse microscopy as the appearance and disappearance of punctate structures. To determine whether Ldb19 was dynamically recruited to the TGN like clathrin adaptors, we performed time-lapse microscopy on cells expressing Ldb19-GFP and Apl4-mCh (Fig 2E). Like Apl4-mCh, Ldb19-GFP was transiently localized to punctate structures. Moreover, consistent with their co-localization in static images, Ldb19-GFP and Apl4-mCh often appeared and disappeared at approximately the same time. Together with the co-localization data, these results suggest that Ldb19 is recruited to the TGN at approximately the same time as AP-1.

**Fig 3:**
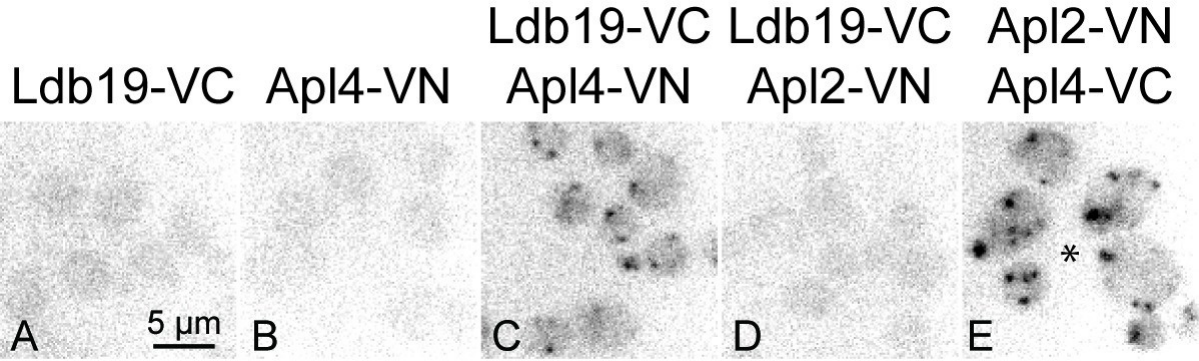
Bimolecular fluorescence complementation (BiFC) demonstrates close localization of Ldb19 with AP-1. Cells expressing indicated fusion proteins at their endogenous loci were imaged for YFP fluorescence. Arrows point to positive BiFC punctate structures in the cells expressing Ldb19-VC and Apl4-VN; asterisk indicates use of diploid strain.

To further explore the relationship between Ldb19 and AP-1, we employed the bimolecular fluorescence complementation (BiFC) technique [34]. BiFC monitors the proximity of two proteins, each tagged with half of a fluorescent protein (e.g. Venus). If the two proteins interact or come into close proximity, the two halves of the protein fold to form a fully fluorescent moiety. Thus, BiFC can show whether two proteins come into close proximity. Given the co-localization of Ldb19 with AP-1, we asked whether Ldb19 comes into close proximity with AP-1 (Fig 3B). We fused Ldb19 with the C-terminal fragment of Venus to generate Ldb19-VC. We tested for proximity to the AP-1 γ-subunit Apl4 and the β-subunit Apl2 fused the N-terminal fragment of Venus (VN). In cells expressing both Ldb19-VC and Apl4-VN, most cells exhibited one to three bright puncta. Curiously, cells expressing both Ldb19-VC and Apl2-VN did not exhibit fluorescence above background level (Fig 3C). This lack of fluorescence was not due to mislocalization or poor expression of Apl2-VN, because cells expressing Apl2-VN and Apl4-VC exhibit BiFC fluorescence (Fig 3D). This specificity may suggest a specific interaction between Ldb19 and the Apl4 subunit of AP-1. However, we were unable to detect an interaction between Ldb19 and AP-1 using immunoprecipitation, suggesting the two may nor interact directly or their interaction may be transient or indirect (unpublished results). Together these results demonstrate that Ldb19 localizes to TGN/endosome regions that also contain AP-1.

**Fig 4:**
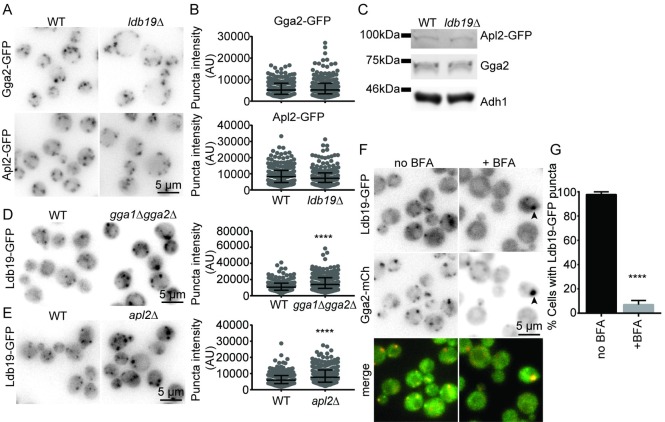
TGN clathrin adaptor localization is unaffected by deletion of *LDB19*. (A) Wild-type and *ldb19Δ* cells expressing Gga2-GFP or Apl2-GFP were imaged. (B) Quantification of the fluorescence intensities of the punctate structures from the images in A. Graphs are of data points measured from a representative experiment showing the median and interquartile ranges (25 and 75 percentile). Each data point corresponds to a single punctate structure. AU, arbitrary units. (C) Western blot showing the protein levels of clathrin adaptor proteins in WT and *ldb19Δ* cells. Adh1 was used as a lysis control. Data is representative of 3 repeats. (D), (E) Wild-type and *gga1Δgga2Δ* or *apl2Δ* cells expressing Ldb19-GFP were imaged. Right, quantification of Ldb19-GFP punctate structures as in B. Non parametric Mann Whitney test was performed (****p > 0.0001). (F) erg6*Δ* cells expressing Ldb19-GFP and Gga2-mCh were imaged following treatment with DMSO (no BFA) or 150μM Brefeldin A (+BFA). Arrowhead points to rare puncta in treated cells. (G) Quantification of the percentage cells from F that contain Ldb19-GFP puncta in the absence or presence of BFA. Non parametric t-test was performed (****p < 0.0001). Graphs from B, D and E were generated from at least 200 punctate structures and at least 70 cells.

Given the close proximity of Ldb19 to AP-1, we next asked whether Ldb19 plays a role in the recruitment of AP-1 or other adaptors to the TGN. To do this, we performed quantitative fluorescent microscopy on cells lacking Ldb19 and expressing GFP-tagged AP-1 or the clathrin adaptor Gga2, which functions at the TGN before AP-1 [32,35]. In cells lacking Ldb19, both Gga2 and AP-1 localized to multiple puncta that appeared similar to the puncta in wild-type cells. Because cells lacking Ldb19 are morphologically abnormal, the distribution of Gga2 and AP-1 looked qualitatively different [8]. This is because cells lacking Ldb19 are large with a large central vacuole. This central vacuole pushes Gga2 and AP-1 containing structures to the periphery (Fig 4). Therefore, to assess the effect of Ldb19 on Gga2 and AP-1 localization, we monitored the intensity of puncta. In cells lacking Ldb19, the intensity of Gga2 and Apl2 structures were not significantly different from those in wild-type cells (Fig 4B). Consistent with limited effect of Ldb19 on the localization and fluorescent intensity of the adaptors, loss of Ldb19 did not alter the protein levels Gga2 or Apl2 as monitored by western blot (Fig 4C). These results argue that Ldb19 does not play a major role in recruiting Gga2 or AP-1.

We next explored how Ldb19 is recruited to the TGN. We first asked whether Gga proteins or AP-1 were important for Ldb19 localization at the TGN. To do this, we performed quantitative fluorescence microscopy in cells lacking the two partially redundant Gga proteins, Gga1 and Gga2, or in cells lacking Apl2. In cells lacking Gga proteins or AP-1, Ldb19 retained its punctate localization (Fig 4D and Fig 4E). Curiously, Ldb19 puncta were significantly brighter in cells lacking the adaptors (Fig 4D and Fig 4E). The cause of the increased intensity is unclear, but could be due to stalled organelle maturation or stalled assembly of specific trafficking complexes leading to enrichment of Ldb19 at the TGN.

Because neither adaptor complex was required for Ldb19 localization, we next asked whether other major regulators of protein localization at TGN contribute to Ldb19 localization. Arf1 is a major regulator of traffic at the TGN [36]. It is required for the association of AP-1 with the TGN, but not for the association of Gga2 or Ent5 [37,38]. We asked whether Ldb19 localization is sensitive to Brefeldin A, a potent inhibitor of Arf1 function at the TGN. In cells treated with Brefeldin A for 5 minutes, Ldb19 largely redistributed to the cytosol (Fig 4F). In most cells, no Ldb19 puncta were visible; however, in a very small fraction of cells (7%) one large puncta was visible (Fig 4G). In contrast, Gga2 was still clearly apparent in punctate structures in most cells, as previously reported [37]. These results suggest Arf1 function is important for Ldb19 localization. The requirement for Arf1 for its localization, together with the close proximity of Ldb19 to AP-1, strongly support the hypothesis that Ldb19 resides on the TGN in the late stages of its maturation, coincident with AP-1.

We next asked whether Ldb19 plays functionally important roles at the TGN. To do this, we asked whether *ldb19Δ* cells show synthetic growth defects when combined with mutations in clathrin or clathrin adaptors at the TGN. Such synthetic growth defects can be potent read-outs of severe trafficking defects. For example, mutations in either AP-1 or Gga2 enhance the growth defect of cells expressing a hypomorphic allele of the clathrin heavy chain (*chc1-ts*) [39–41]. Similarly, mutations in AP-1 and Ggas are synthetic lethal with one another or synthetic sick depending on the specific alleles combined [42,43].

**Fig 5:**
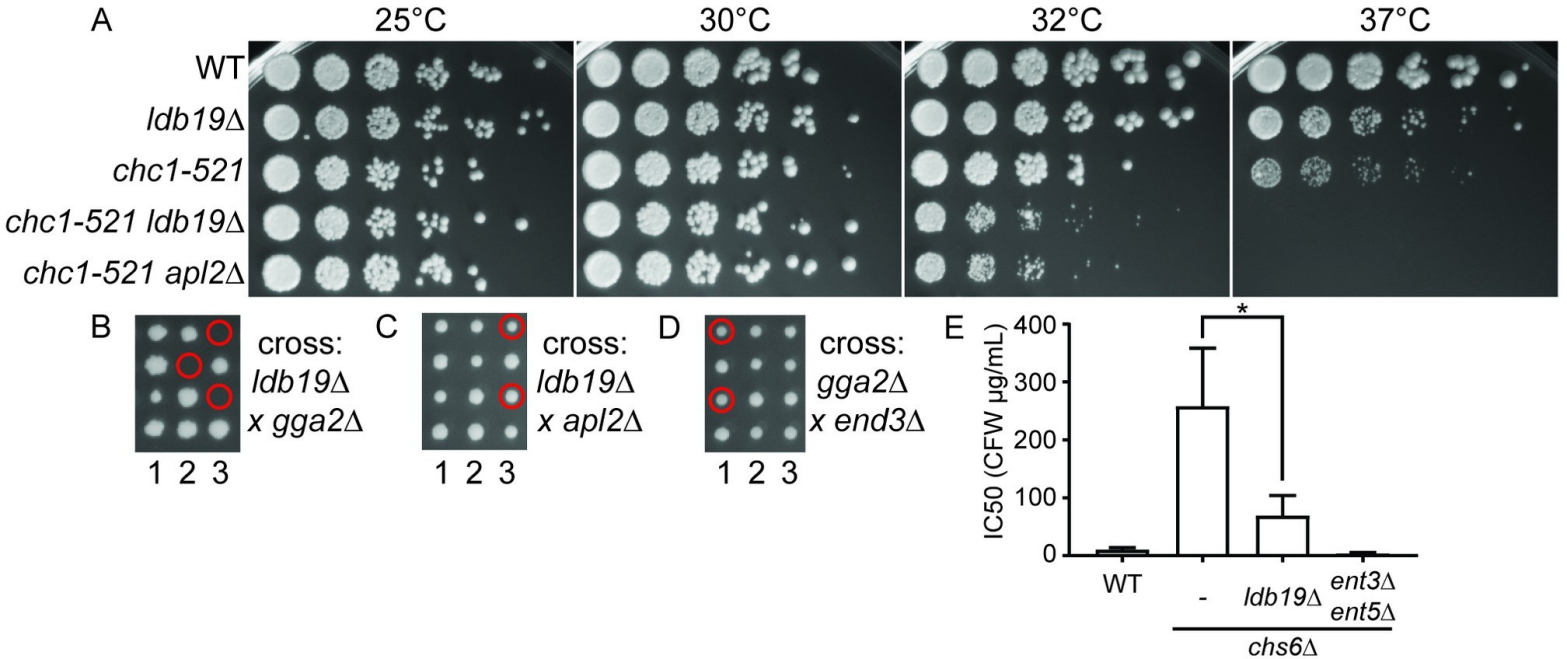
Deletion of *LDB19* increases temperature sensitivity of *chc1-ts* and is synthetic lethal with *GGA2*. (A) Serial dilutions of the indicated strains were spotted on YPD plates and grown for three days at the indicated temperatures. (B) Tetrad dissection plates from a cross between l*db19Δ* and *gga2Δ* strains. (C) Tetrad dissection from a cross between *ldb19Δ* and *apl2Δ* strains. (D) Tetrad dissection from a cross between *end3Δ* and *gga2Δ* strains. Red circles indicate colonies with double deletion genotypes deduced either by direct genotyping or by inference from viable siblings of inviable spores. (E) Calcofluor white sensitivity was determined using a quantitative growth analysis approach in indicated strains.

To test whether *LDB19* has genetic interactions similar to AP-1 or *GGA2*, we first tested whether deletion of *LDB19* enhanced the *chc1-ts* growth defect. Loss of *LDB19* strongly enhanced the growth defect caused by the *chct1-ts* allele. At 37°C, *chc1-ts* cells grew slowly, however the double mutant cells did not grow at all (Fig 5A). Similarly, at 32°C, *chc1-ts* cells grow nearly as well as wild-type cells whereas the double mutant grew much more poorly. This growth defect was similar to the growth defect caused by loss of AP-1 in cells carrying the *chc1-ts* allele. This genetic interaction is consistent with a role for Ldb19 at the TGN. We next asked whether *LDB19* shows genetic interactions with AP-1 or *GGA2*. To do this, we generated diploid cells by crossing *ldb19Δ* cells with cells lacking either *GGA2* or *APL2*. We then induced sporulation, separated meiotic progeny of single diploid cells using a micromanipulator, and monitored the growth of the colonies formed from the haploid spores. In crosses between cells carrying deletions of *LDB19* and *GGA2*, approximately one quarter of the spores were inviable. This is the segregation pattern expected from unlinked synthetic lethal genes. Genotyping revealed that none of the viable spores contained deletion of both *LDB19* and *GGA2*. Moreover, the genotype of the inviable spores could be deduced by the genotype of the viable siblings (Fig 5B, red circle). This analysis revealed that the inviable cells carried deletion of both *LDB19* and *GGA2*, indicating the two deletions are synthetic lethal as previously reported (Fig 5B and [44]). In contrast, *ldb19Δ* was not synthetic lethal with *apl2Δ* because double mutant cells were identified in the correct proportion from the cross between cells lacking *LDB19* and *APL2*, and the double mutant cells grew as well as their wild-type siblings. This indicates that the synthetic lethality between *ldb19Δ* and *gga2Δ* was highly specific. Because Ldb19 is known to function in endocytosis, we asked whether the synthetic lethality between *ldb19Δ* and *gga2Δ* could be explained by the endocytic functions of Ldb19. To do this we crossed cells carrying deletion *END3*, an endocytic gene, to cells lacking *GGA2*. *GGA2* was not synthetic lethal with *END3* (Fig 5D). These data suggest that the synthetic lethality between *LDB19* and *GGA2* is not due to the role of Ldb19 in endocytosis and could suggest a role for Ldb19 in AP-1 dependent traffic at the TGN.

**Fig 6:**
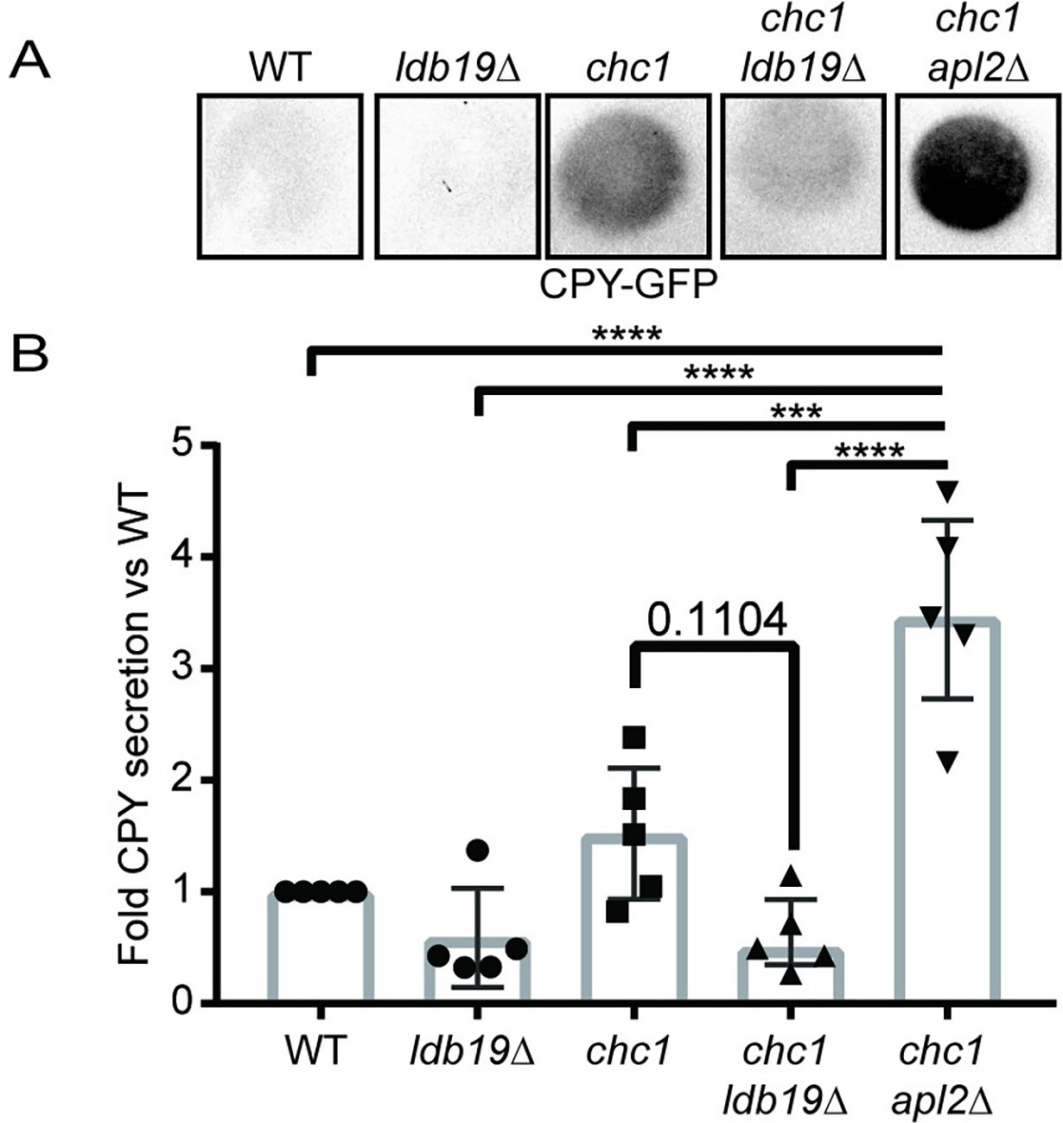
Deletion of *LDB19* does not enhance chc1-ts CPY secretion. (A) Colony immunoblot to monitor CPY secretion of the indicated strains. Shown are a representative immunoblots. (B) Corresponding quantification for the data in A by densitometry analysis. Bar graphs are of the median with interquartile range with data points shown (n = 5). Data were analyzed by one-way ANOVA. p-values: *** = <0.005, **** = <0.001. Full-length blots are presented in S1 Fig.

To further examine the role of Ldb19 at the TGN, we monitored the effect of Ldb19 on the calcofluor white sensitivity of cells lacking Chs6. Chs6 is a component of a coat that mediates the transport of the chitin synthase Chs3 from the Golgi to the plasma membrane [45]. Cells lacking Chs6 are resistant to calcofluor white due to reduced levels of Chs3 at the cell surface, which reduces the binding of calcofluor white to the cell wall. Mutations that disrupt clathrin mediated traffic at the TGN partially restore calcofluor white sensitivity [46–48]. This phenotype is thought to result from defective retention of Chs3 in the TGN or defective recycling from the endosome. Deletion of *LDB19* increased the sensitivity of cells lacking Chs6 to calcofluor white, suggesting a potential role in the retention or recycling of Chs3, similar to the role reported for clathrin-dependent traffic at the TGN (Fig 5E). We next examined secretion of CPY, a vacuolar protease that normally is efficiently sorted at the TGN for traffic to the vacuole [49]. In cells with strong defects in clathrin-dependent traffic at the TGN, CPY is mis-sorted to the plasma membrane and is secreted [37]. Cells carrying the *chc1-ts* allele secrete CPY. These effects are strongly enhanced by loss of AP-1. To monitor CPY secretion, we performed colony immunoblots using cells that express CPY-GFP (Fig 6 and S1 Fig). As expected, in WT cells and cells lacking only *LDB19* or *APL2*, secreted CPY was not detected. In contrast, cells carrying the *chc1-ts* allele secreted CPY resulting in weakly detectable CPY signal. This defect was enhanced by deletion of *APL2*. Surprisingly, although the *ldb19Δ chc1-ts* double mutant grew as slowly as the *apl2Δ chc1-ts* double mutant, the *ldb19Δ chc1-ts* had less CPY signal than cells carrying the *chc1-ts* allele alone. This indicates that loss of *LDB19* does not cause the same effect on CPY sorting as loss of AP-1.

Because *ldb19Δ* and *apl2Δ* showed dissimilar phenotypes for CPY secretion when combined with the *chc1-ts* allele, we performed an alternative test of clathrin function at the TGN. To do this, we monitored the secretion of unprocessed α-factor using colony immunoblot. α-factor is a secreted hormone involved in yeast cell mating. At the TGN, α-factor is cleaved from its high-molecular weight pro-form to the mature peptide hormone [49]. This cleavage is mediated by Kex2, a furin-like protease whose steady state localization at the TGN is disrupted in cells with strong defects in clathrin function at the TGN [40,50]. When Kex2 localization is disrupted, unprocessed α-factor is secreted. This unprocessed α-factor, but not the mature form, adheres strongly to nitrocellulose allowing its detection by colony immunoblot [51]. We found that cells carrying the *chc1-ts* secreted substantial levels of unprocessed α-factor and this effect was enhanced by deletion of *APL2* (S2 Fig). Similar to the effect on CPY, the α-factor signal was lower in the *ldb19Δ chc1-ts* double mutant than cells carrying the *chc1-ts* allele alone (S2 Fig). These results suggest that despite their close proximity, similar requirements for localization, and similar genetic interactions, Ldb19 may not be required for AP-1 mediated traffic of CPY and Kex2.

## Discussion

Here we provide several lines of evidence that Ldb19 localizes at the TGN, confirming prior reports [22,25]. Although a small proportion of Ldb19 was visible at the plasma membrane, the majority of Ldb19-GFP strongly co-localized with markers of the TGN. Our results extend prior findings about Ldb19 localization by positioning Ldb19 within the dynamic lifespan of the TGN. The TGN is now thought to be a dynamic organelle that continually forms, matures, and dissipates due to repeated cycles of traffic [28,29]. In this maturation cycle, AP-1 labels a late stage [32,35]. Our results indicate that Ldb19 is preferentially associated with these late-stage, AP-1 positive TGN compartments. In fluorescence microscopy experiments, AP-1, a marker of late stages, co-localized more strongly with Ldb19 than did Gga2, a marker of earlier stages. Furthermore, in BiFC experiments, Ldb19 came into close proximity to AP-1 and like AP-1, Ldb19 localization depends on the same GTPase Arf1. Although our results do not preclude the possibility that Ldb19 also localizes to other locations or is recruited to the TGN prior to the late stages, these results strongly suggest that Ldb19 is present on late stage TGN in close proximity to AP-1.

The function of Ldb19 at this location remains elusive. Some of the phenotypes caused by loss of Ldb19 are similar to those caused by loss of AP-1, including the genetic interactions with *chc1-ts* and *gga2Δ*, and effect on calcofluor white sensitivity. This might indicate Ldb19 cooperates with AP-1 to mediate traffic of some cargo at the TGN. However, the effects of *ldb19Δ* on the secretion of CPY and processing of α-factor differ substantially from the effects caused by deletion of AP-1 subunits, suggesting Ldb19 may selectively modify AP-1 mediated traffic at the TGN. Regardless of the exact functions at the TGN, our results strongly argue that Ldb19 functions at the TGN, in addition to its well-established functions at the plasma membrane.

The possibility that Ldb19 functions at the TGN is notable in light of recently published work, which indicates that Ldb19 disrupts traffic at internal compartments. In one recent study, overexpression of Ldb19 enhanced the cell surface levels of an ectopically expressed mammalian potassium channel protein, Kir2.1 [52]. This phenotype indicates that Ldb19 functions in the recycling or biosynthetic delivery of Kir2.1 to the plasma membrane. In addition, Ldb19 is required for post-endocytic traffic of the yeast arginine permease, Can1 [23]. In this case, in cells lacking Ldb19, the endocytosis of Can1 was unaffected. However, Can1 did not reach the vacuole, suggesting that Ldb19 promotes traffic of Can1 at internal compartments. This finding is significant, because the TGN is the departure point for newly synthesized proteins and serves as the early endosome in yeast [35]. Therefore, our finding that functional Ldb19 localizes at the TGN suggests that both of these unexpected phenotypes may be due to a direct role of Ldb19 in controlling the traffic of Kir2.1 and Can1 at the TGN.

Our finding that Ldb19 has functions at both the TGN and plasma membrane expands the list of ART proteins that function at multiple compartments and raise the possibility that this is a general feature of the ART family. To date, the ARTs Bul1, Bul2, Aly1, Aly2, and Rod1 have been implicated in functions at both the plasma membrane and internal compartments [12,19,53–55]. Adding Ldb19 to this list expands the list of ARTs that function at multiple compartments to 6 out of 12. Notably, these six ART proteins occupy all branches of the ART family identified by sequence homology [8,10]. Based on sequence homology, Bul1&2 form their own subfamily, Aly1, Aly2 and Ldb19 belong to a subfamily that includes a pair of highly similar ARTs (Csr2/Art8, Ecm21/Art2), whereas Rod1 resides on the third branch that contains 3 additional ART proteins. It is therefore likely that the common progenitor of the gene family was capable of functioning at multiple compartments, and suggests that this multi-functionality may be a general feature of all ART proteins. Indeed multi-functionality appears to be a facet of ARTs in distantly related yeast, supporting this possibility [14].

Although Ldb19 strongly associates with the TGN, the direct molecular mechanism that recruits Ldb19 to the TGN is unclear. Despite close proximity to AP-1, as monitored by BiFC, Ldb19 does not depend on AP-1 for localization to punctate structures. We found that Ldb19 localization was sensitive to Brefeldin A, suggesting that it depends on Arf for localization. It remains to be determined whether Ldb19 directly interacts with Arf or whether this is an indirect effect. Notably, Ldb19 has broad affinity for small GTPases [56]. In GST-pulldown experiments, Ldb19 bound to three unrelated GTPases Ypt1, Ras2, and Rho1, raising the possibility that Ldb19 may interact directly with Arf1. Alternatively, Ldb19 could interact with other GTPases that localize to the TGN including Ypt31, Ypt32, and Rho3 [57,58]. Future work is necessary to understand whether these interactions control Ldb19 localization at the TGN, and whether similar interactions direct the intracellular localization of other ART proteins.

## Materials and methods

### Reagents, strains, growth conditions

Rabbit polyclonal antibodies against full-length Gga2, Ent5 and α-factor have been previously described [59,60]. Rabbit polyclonal Adh1 (RRID: RRID:AB_722702) were from Abcam (Cambridge, MA), mouse monoclonal GFP antibodies (RRID: AB_627695) were from Santa Cruz Biotechnology (Santa Cruz, CA), goat anti-rabbit (RRID: AB_141663) and goat anti-mouse (RRID:AB_141698) Alexa Fluor 647 secondary antibodies were from Invitrogen (Carlsbad, CA), goat anti-rabbit HRP-conjugated secondary antibodies (RRID:AB_258167) were from Invitrogen (Carlsbad, CA).

Fluorescent tags, BiFC tags and gene deletions were introduced by standard PCR-based procedures [61,62] in the *S*. *cerevisiae* BY4743 background and its progeny. Strains containing multiple genomic modifications were generated by standard yeast genetics.

Yeast cells were grown at 30°C and aerated by rotary shaking. For non-selective growth, cells were grown in Yeast peptone (YPD) media (1% bacto-yeast extract and 2% bacto-peptone (Difco, Detroit, MI) supplemented with 2% dextrose and 20 μg/ml adenine, uracil, and L-tryptophan. For selective conditions, cells were grown in Synthetic defined (SD) media (0.67% of yeast nitrogen base without amino acids (Difco) and 2% dextrose. Supplemented SD media contained 100μg/ml adenine, L-leucine, L-lysine, L-tryptophan; 50 μg/ml L-histidine, L-methionine, and 20 μg/ml uracil) or in media lacking L-methionine for the expression of Mup1-GFP. Sporulation of diploid cells was performed by incubation of saturated cultures for 4 days in sporulation media (10mg/mL potassium acetate, 1mg/mL yeast extract and 0.5mg/mL dextrose) supplemented with 10μg/ml adenine, L-leucine, L-lysine, L-tryptophan; 5 μg/ml L-histidine, L-methionine, and 2 μg/ml uracil.

For Mup1-GFP internalization experiments, cells were grown to mid-log phase in SD media lacking methionine. To induce internalization of Mup1, cells were resuspended in SD media containing methionine for 1hr prior to imaging. For Brefeldin A experiments, cells were treated with a final concentration of 150μM Brefeldin A (Acros Organics, New Jersey) for 5 minutes prior to imaging.

To determine changes in growth of *chc1-ts* expressing cells when combined with *ldb19Δ*, 200 μl of log phase cells (OD_600_ = 0.5) were transferred into wells of a 96-well plate. The cultures were serially diluted 5-fold in adjacent wells. The cells were replica pinned onto agar plates using a 48-well solid pin tool (Sigma, St. Louis, MO). The plates were incubated at room temperature until the liquid media was absorbed into the agar. The plates were incubated at indicated temperatures for 2–3 days before imaging the plates. Experiments were repeated 3 times using two different strains for each genotype.

To monitor synthetic lethal phenotypes, diploid cells were generated by standard methods and sporulation was induced by transferring into sporulation media and culturing at 25°C. When fully formed tetrad were visible (3–5 days), tetrads were dissected onto YPD plates using a microscope-mounted micromanipulator. Plates were incubated at 30°C until colonies were visible.

Calcofluor white sensitivity was performed as previously described [63].

### Immunoblotting

For immunoblots of cell extracts, cell extracts were prepared from 2 OD_600_ of cells. Extracts were generated by resuspending in Laemmli lysis buffer [64], boiling, and vortexing in the presence of glass disruption beads. The extracts were cleared by centrifugation. Proteins were separated by SDS–PAGE, transferred to nitrocellulose, blocked with 5% milk in Tris-buffered saline with Tween and then probed with primary (1:25,000 dilution for anti-Adh1, 1:3,000 dilution for all other antibodies) and fluorescent or HRP secondary antibodies (1:20,000 dilution). The Immobilon western chemiluminescent HRP substrate use for chemiluminescence was from Millipore (Millipore, Billerica, MA). Fluorescence or chemiluminescence signal was detected on an Azure c600 imaging system (Azure Biosystems, Dublin, CA).

For colony immunoblots, yeast cultures grown in YPD to mid-log phase (0.3–0.5 OD_600_) were diluted to 0.1 OD_600_ and 5μL were transferred onto a YPD plate. Plates were incubated at 30°C overnight (16-18hr) to allow growth. To capture secreted proteins, a piece of nitrocellulose was first wetted with sterile water and placed on top of the YPD plate. The plate was incubated for a further 8-12hr at room temperature (25°C). The nitrocellulose membrane was removed and adhered cells were removed by rinsing with water. The nitrocellulose membrane was then immunoblotted with primary antibodies and secondary antibodies as described for cell extracts.

### Microscopy

For microscopy, cells were grown to log phase in SD supplemented with appropriate amino acids mixes, and then briefly pelleted and resuspended to concentrate prior to imaging. Images for Fig 2 were captured using a DeltaVision^TM^ Elite system (GE Healthcare Life Sciences), equipped with an Olympus IX-71 inverted microscope, a sCMOS CAMERA, A 100x/1.4 Oil Super-Plan APO objective, and a DeltaVision Elite Standard Filter Set with the FITC filter (excitation:475/28, emission:525/48) for GFP and the TRITC filter (excitation: 542/27, emission: 594/45) for mCh. Image acquisition and deconvolution were performed with the program softWoRx. All other fluorescence imaging, was performed on a Nikon Ti-E inverted microscope with a 1.4 numerical aperture, 100× oil-immersion objective and an Andor iXon3 EMCCD camera. A Lumencor LED light engine was used for fluorophore excitation. Image acquisition was performed the MetaMorph acquisition software. For each field, 10-slice Z-stacks (0.25 μm apart) were captured. For image cropping and other figure preparation steps, the ImageJ version 1.50i (National Institutes of Health) software was used.

### Image processing and imaging analysis

The intensity of fluorescence puncta in cells was quantified as follows: Z-stack images were first compressed to a single maximum-intensity image using ImageJ version 1.50i. The resulting image was denoised using the “Denoise image” menu from the SpatTrackV2 software [65]. To define regions of GFP localization, the denoised image was thresholded using Otsu’s method with ImageJ with the stacked histogram option selected. GFP positive regions of interest were defined using the “Analyze Particles” menu from ImageJ on the binary thresholded image. The GFP positive regions of interest were then used to measure fluorescence intensity from the original, compressed non-denoised Z-stack. Fluorescence intensity was determined from at least 100 cells and 200 total number of puncta. Experiments were repeated 3 times using at least two different strains for the same genotype.

For co-localization analysis, GFP and mCherry positive regions of interest were defined using the same process used for puncta intensity analysis. GFP structures that contain mCherry, were defined as structures for which at least 20% of the GFP positive area of the structure was also mCherry positive and vice versa for mCherry structures that contain GFP. Fluorescence intensity was determined from at least 100 cells and 200 total number of puncta. Experiments were repeated 3 times using two different strains for each genotype. Graphs were created using GraphPad Prism v7.00 (GraphPad Software, La Jolla, CA).

## Supporting information

S1 Fig Original positions of dot blots in raw images for Fig 6. Top row, dot blot rearranged as in Fig 6; bottom row, dot blot before rearrangements (original) for Fig 6. Arrows connect each dot from its rearranged (top) to its original positions (bottom). The genotypes of controls not used for the final figure are shown.
(TIF)

S2 Fig α-factor colony immunoblot. (A) Colony immunoblot to test α-factor secretion of the indicated strains. Shown are a representative immunoblot and the corresponding quantification by densitometry analysis. Colony spots come from the same immunoblot, but rearranged for clarity. (B) Original image of colony immunoblots shown in A.
(TIF)

S3 Fig Western blot membranes used in Fig 4C. Shown are uncropped images of the membrane used in Fig 4C. The top image was used to show levels of Apl2-GFP, the middle to show levels of Gga2 and the bottom to show levels of Adh1. All three images are from the same membrane, at different exposures.
(TIF)

S1 Table *Saccharomyces cerevisiae* strains used in this study. (PDF)

S1 Movie Time-lapse imaging of Apl4-mCh and Ldb19-GFP. Time-lapse movie of single cell expressing Apl4-mCh (left) and Ldb19-GFP (middle). Color merge time-lapse video is also shown (right). Time-lapse movie is 91 frames long with a speed of 7 frames per second.
(AVI)
