# Supplementary figures and images for: Investigation of Ldb19/Art1 localization and function at the late Golgi
Source: PLoS One. 2018 Nov 7;13(11):e0206944. doi: 10.1371/journal.pone.0206944 (PMC6221343; doi:10.1371/journal.pone.0206944)

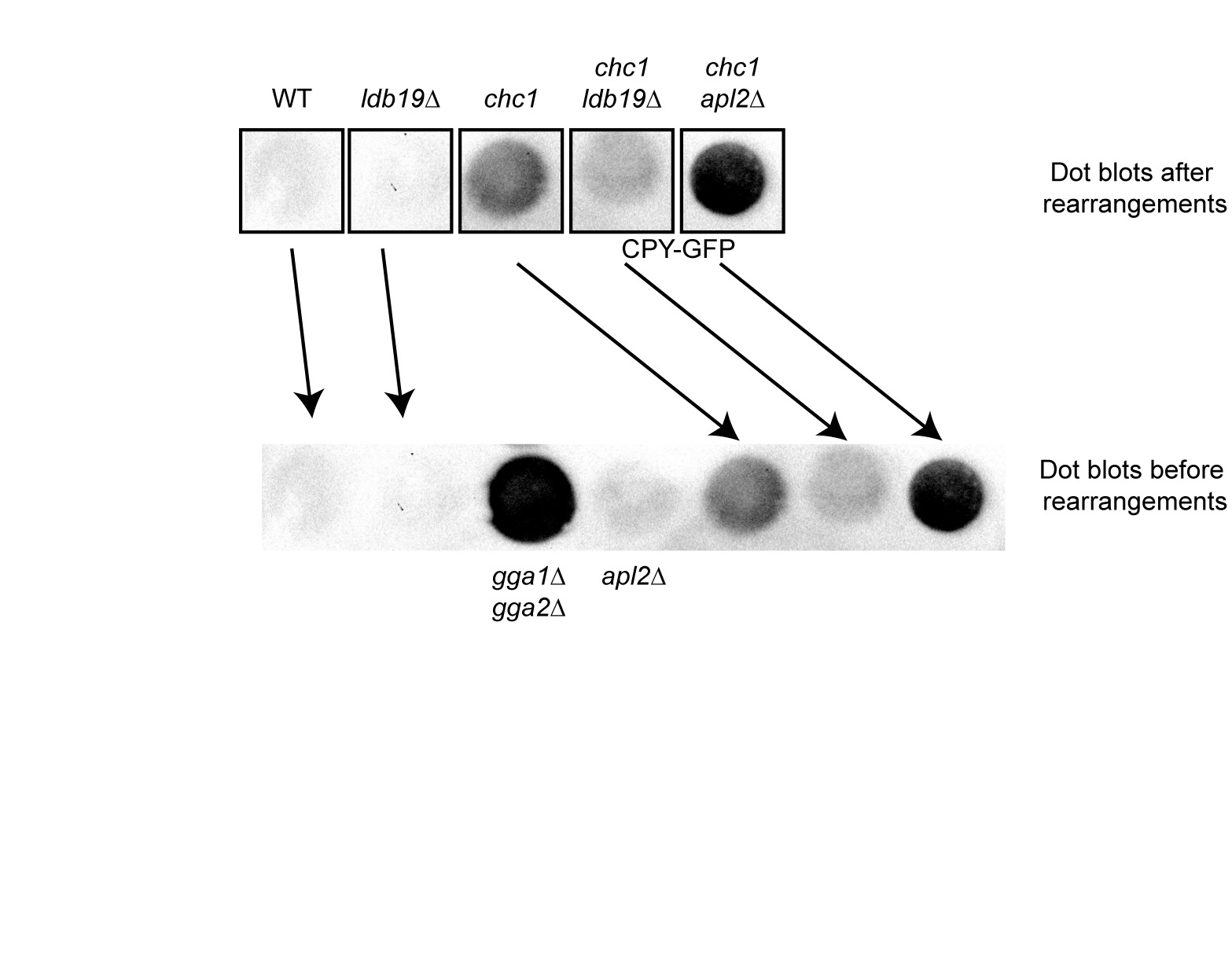

Supplement: S1 Fig — Top row, dot blot rearranged as in Fig 6; bottom row, dot blot before rearrangements (original) for Fig 6. Arrows connect each dot from its rearranged (top) to its original positions (bottom). The genotypes of controls not used for the final figure are shown. (TIF) [file pone.0206944.s001.tif]

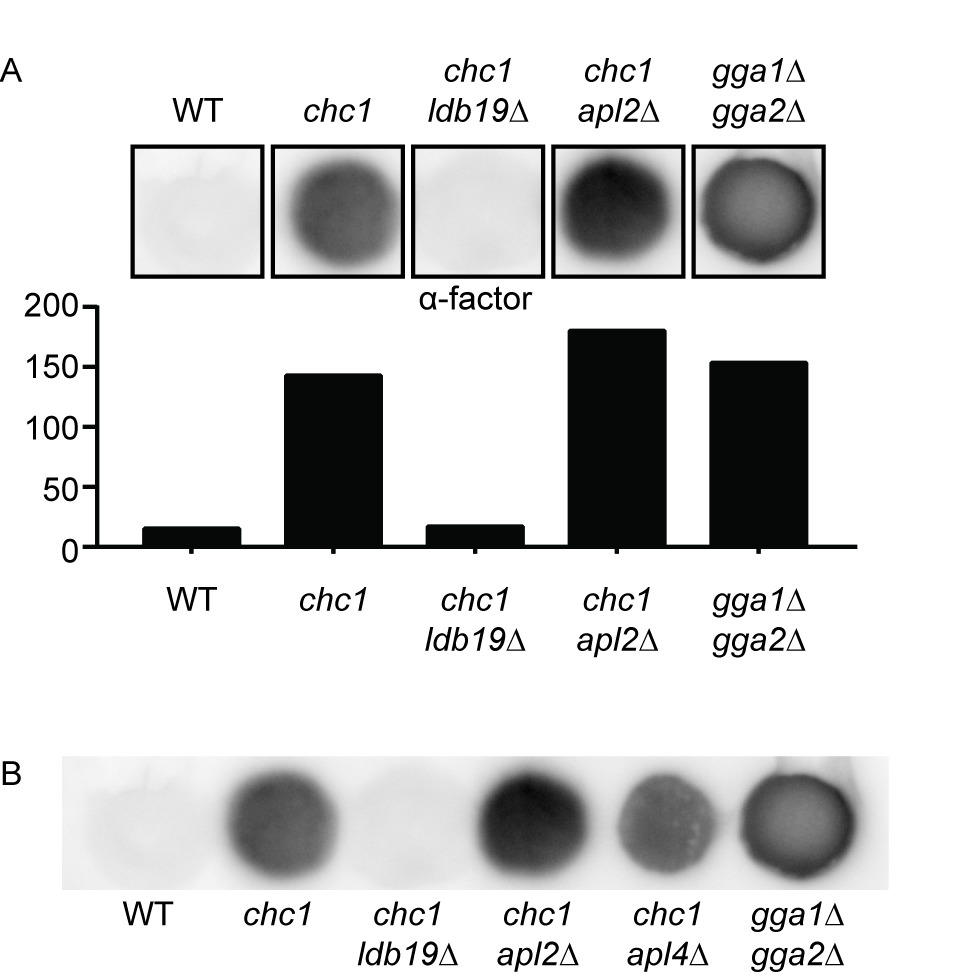

Supplement: S2 Fig — (A) Colony immunoblot to test α-factor secretion of the indicated strains. Shown are a representative immunoblot and the corresponding quantification by densitometry analysis. Colony spots come from the same immunoblot, but rearranged for clarity. (B) Original image of colony immunoblots shown in A. (TIF) [file pone.0206944.s002.tif]

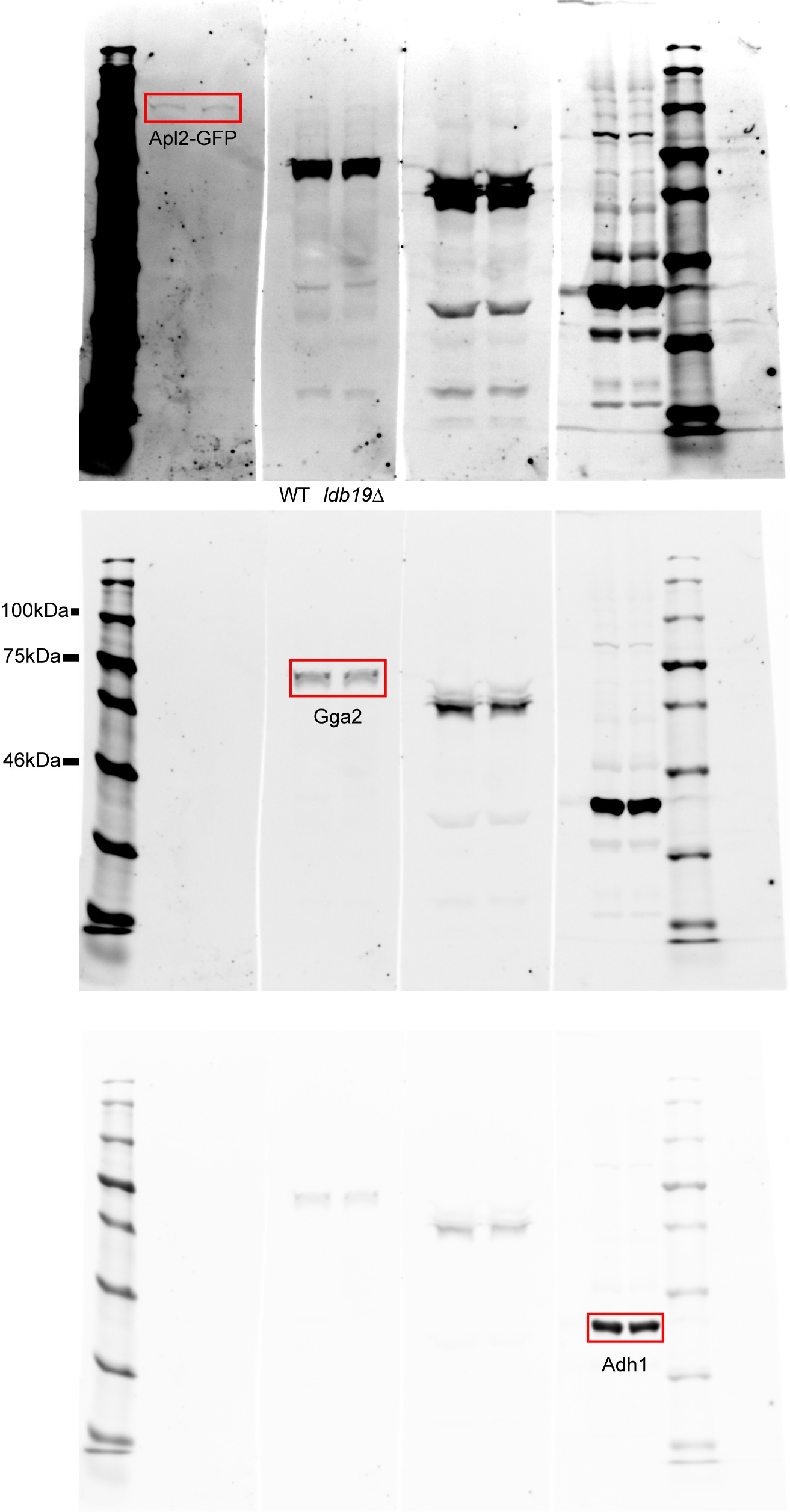

Supplement: S3 Fig — Shown are uncropped images of the membrane used in Fig 4C. The top image was used to show levels of Apl2-GFP, the middle to show levels of Gga2 and the bottom to show levels of Adh1. All three images are from the same membrane, at different exposures. (TIF) [file pone.0206944.s003.tif]
