# Supplementary material for: Investigation of Ldb19/Art1 localization and function at the late Golgi
Source: PLoS One. 2018 Nov 7;13(11):e0206944. doi: 10.1371/journal.pone.0206944 (PMC6221343; doi:10.1371/journal.pone.0206944)
Supplement: S1 Table — (PDF) [file pone.0206944.s004.pdf]

Supplementary information

Investigation of Ldb19/Art1 localization and function at the late Golgi

Jorge Y. Martínez-Márquez and Mara C. Duncan

Supplementary Table S1: *Saccharomyces cerevisiae* strains used in this study.

Strain	Genotype	Source
BY4743	*Mat a his3Δ1/his3Δ1 leu2Δ0/leu2Δ0 ura3Δ0/ura3Δ0 met15Δ0/MET15 LYS2/lys2Δ0*	Brachmann et al., 1998
DLY1685	*Mat α his3Δ1 leu2Δ0 ura3Δ0 MET15 lys2Δ0 ldb19Δ::KanMx6 Mup1-GFP::HisMx6*	This study
DLY2020	*Mat a his3Δ1 leu2Δ0 ura3Δ0 MET15 LYS2*	Hung et al., 2018
DLY1891	*Mat a his3Δ1 leu2Δ0 ura3Δ0 MET15 LYS2 ldb19Δ::KanMx6*	This study
DLY740	*Mat a his3Δ1 leu2Δ0 ura3Δ0 met15Δ0 LYS2 Ldb19-GFP::HisMx6*	This study
DLY1103	*Mat a his3Δ1 leu2Δ0 ura3Δ0 MET15 lys2Δ0 Ldb19-GFP::HisMx Gga2-mCh::KanMx*	This study
DLY1105	*Mat a his3Δ1 leu2Δ0 ura3Δ0 MET15 LYS2 Ldb19-GFP::HisMx Ent5-mCh::KanMx*	This study
DLY3028	*Mat a his3Δ1 leu2Δ0 ura3Δ0 met15Δ0 lys2Δ0 Ldb19-GFP::HisMx Apl4-mCh::HisMx*	This study
DLY1458	*Mat α his3Δ1 leu2Δ0 ura3Δ0 met15Δ0 LYS2 Ldb19-GFP::HisMx Vps35-mCh::HisMx*	This study
DLY1914	*Mat α his3Δ1 leu2Δ0 ura3Δ0 MET15 lys2Δ0 Ldb19-VC::HisMx*	This study
DLY3466	*Mat a his3Δ1 leu2Δ0 ura3Δ0 MET15 lys2Δ0 Ldb19-VC::HisMx Apl4-VN::HisMx*	This study
DLY3612	*Mat α his3Δ1 leu2Δ0 ura3Δ0 MET15 lys2Δ0 Ldb19-VC::HisMx Apl2-VN::HisMx*	This study
DLY3348	*Mat a his3Δ1 leu2Δ0 ura3Δ0 MET15 LYS2 Apl4-VN::HisMX*	This study
DLY3344	*Mat α his3Δ1 leu2Δ0 ura3Δ0 MET15 LYS2 Apl2-VN::HisMx*	This study
DLY3346	*Mat a his3Δ1 leu2Δ0 ura3Δ0 MET15 LYS2 Apl4-VC::HisMX*	This study
DLY929	*Mat a his3Δ1 leu2Δ0 ura3Δ0 MET15 lys2Δ0 Gga2-GFP::KanMX*	This study
DLY1710	*Mat a his3Δ1 leu2Δ0 ura3Δ0 MET15 lys2Δ0 ldb19Δ::KanMx Gga2-GFP::KanMx*	This study
DLY2669	*Mat a his3Δ1 leu2Δ0 ura3Δ0 MET15 LYS2 Apl2-GFP::HisMx*	This study
DLY3063	*Mat a his3Δ1 leu2Δ0 ura3Δ0 met15Δ LYS2 ldb19Δ::KanMx Apl2-GFP::HisMx*	This study
DLY3555	*Mat α his3Δ1 leu2Δ0 ura3Δ0 MET15 LYS2 erg6Δ::KanMx Ldb19-GFP::HisMx Gga2-mCh::HisMx*	This study
DLY3004	*Mat a his3Δ1 leu2Δ0 ura3Δ0 met15Δ0 LYS2 Ldb19-GFP::HisMx gga1Δ::HisMx gga2Δ::KanMx*	This study
DLY3764	*Mat a his3Δ1 leu2Δ0 ura3Δ0 MET15 LYS2 Ldb19-GFP::HisMx apl2Δ::KanMx*	This study
MDY650	*Mat α his3Δ1 leu2Δ0 ura3Δ0 MET15 lys2Δ0 chc1-ts::Ura3Mx*	This study
DLY2904	*Mat a his3Δ1 leu2Δ0 ura3Δ0 met15Δ0 LYS2 ldb19Δ::KanMx chc1-ts::Ura3Mx*	This study
DLY253	*Mat α his3Δ1 leu2Δ0 ura3Δ0 MET15 lys2Δ0 apl2Δ::KanMX chc1-ts::Ura3Mx*	This study
DLY254	*Mat α his3Δ1 leu2Δ0 ura3Δ0 met15Δ0 LYS2 gga2Δ::KanMX*	This study
DLY2857	*Mat a his3Δ1 leu2Δ0 ura3Δ0 MET15 lys2Δ0 apl2Δ::KanMX*	This study
DLY801	*Mat a his3Δ1 leu2Δ0 ura3Δ0 met15Δ0 LYS2 end3Δ::KanMx Tat1-GFP::HisMx*	This study
DLY29	*Mat α his3Δ1 leu2Δ0 ura3Δ0 MET15 lys2Δ0 chs6Δ::KanMx*	Invitrogen
DLY248	*Mat α his3Δ1 leu2Δ0 ura3Δ0 MET15 lys2Δ0 chs6Δ::KanMx*	Hung & Duncan 2016
DLY1947	*Mat a his3Δ1 leu2Δ0 ura3Δ0 MET15 lys2Δ0 chs6Δ::KanMx ldb19Δ::KanMx*	This study
DLY1948	*Mat α his3Δ1 leu2Δ0 ura3Δ0 met15Δ0 lys2Δ0 chs6Δ::KanMx ldb19Δ::KanMx*	This study
DLY2350	*MATα leu2-3,112 ura3-52 his3-Δ200 trp1-Δ901 suc2-Δ9 lys2-801; GAL chs6Δ::TRP1 ent3Δ::TRP1 ent5Δ::TRP1*	Hung et al., 2018
CWY110	*MATα leu2-3,112 ura3-52 his3-Δ200 trp1-Δ901 suc2-Δ9 lys2-801; GAL chs6Δ::TRP1 ent3Δ::TRP1 ent5Δ::TRP1*	This study
DLY2021	*Mat α his3Δ1 leu2Δ0 ura3Δ0 MET15 LYS2*	This study
DLY2906	*Mat α his3Δ1 leu2Δ0 ura3Δ0 met15Δ0 lys2Δ0 ldb19Δ::KanMx chc1-ts::Ura3Mx*	This study

DLY250	*Mat α his3Δ1 leu2Δ0 ura3Δ0 MET15 lys2Δ0 apl4Δ::KanMX chc1-ts::Ura3Mx*	This study
DLY2614	*Mat α his3Δ1 leu2Δ0 ura3Δ0 MET15 LYS2 gga1Δ::HisMx gga2Δ::KanMx*	This study
DLY3722	*Mat a his3Δ1 leu2Δ0 ura3Δ0 met15Δ0 LYS2 CPY-GFP::HisMx*	Huh et al., 2003
DLY3771	*Mat α his3Δ1 leu2Δ0 ura3Δ0 met15Δ0 LYS2 CPY-GFP::HisMx gga1Δ::HisMx gga2Δ::KanMx*	This study
DLY3774	*Mat a his3Δ1 leu2Δ0 ura3Δ0 met15Δ0 LYS2 CPY-GFP::HisMx apl2Δ::KanMx*	This study
DLY3779	*Mat α his3Δ1 leu2Δ0 ura3Δ0 met15Δ0 lys2Δ0 CPY-GFP::HisMx chc1-ts::Ura3Mx*	This study
DLY3781	*Mat a his3Δ1 leu2Δ0 ura3Δ0 MET15 LYS2 CPY-GFP::HisMx chc1-ts::Ura3Mx ldb19Δ::KanMx*	This study
DLY3777	*Mat α his3Δ1 leu2Δ0 ura3Δ0 MET15 lys2Δ0 CPY-GFP::HisMx chc1-ts::Ura3Mx apl2Δ::KanMx*	This study
